# A Novel Approach for Underwater Vehicle Localization and Communication Based on Laser Reflection

**DOI:** 10.3390/s19102253

**Published:** 2019-05-15

**Authors:** Shijun Wu, Puzhe Zhou, Canjun Yang, Yushi Zhu, Hui Zhi

**Affiliations:** State Key Laboratory of Fluid Power and Mechatronic Systems, Zhejiang University, Hangzhou 310027, China; bluewater@zju.edu.cn (S.W.); zhuyushi@zju.edu.cn (Y.Z.); cheyir63278@163.com (H.Z.)

**Keywords:** laser reflection, corner cube retroreflector, tracking, localization, communication, modulating retroreflector

## Abstract

This study presents a device for tracking, locating and communicating underwater vehicles as they work near the seabed. The system includes a base station placed on the seabed and a reflective module mounted on a hybrid underwater profiler (HUP). The base station localizes and communicates with the HUP working near the seabed based on laser reflections of corner cube retroreflectors. A tracking method based on the particle filter algorithm is then presented. Localization is performed using the least-squares method with refraction compensation. Lost tracking links are retrieved via a recovering approach based on the interpolation method. Finally, a communication method using a modulating retroreflector installed on the reflection module is proposed. The proposed tracking, localization, and communication approach provides higher localization accuracy with lower power consumption at low cost compared with the commonly used acoustic methods. The effectiveness of the proposed approach was clarified via tracking, localization, and communication experiments.

## 1. Introduction

With the expansion of ocean exploration, an increasing number of underwater vehicles are being deployed in marine scientific research and resource prospecting. Such vehicles include autonomous underwater vehicles (AUVs), remotely operated underwater vehicles (ROVs), underwater gliders, and profilers [1,2,3,4]. Localization and communication ability are among the most important requirements of underwater vehicles. Localization provides accurate position information for navigation and accurate spatial information for oceanographic data [5,6], whereas communication ensures that underwater vehicles can transmit the collected data while receiving command information [7,8]. However, the localization and communication of underwater vehicles remain imperfect, particularly in close-range and near-seabed situations.

Because electromagnetic waves are attenuated rapidly in seawater, typical device to device communication and localization methods used on land cannot be directly applied to underwater equipment [9]. Therefore, most underwater vehicles realize localization and communication using the acoustic method [10,11,12]. Although sound waves can propagate underwater over long distances with little attenuation [13], acoustic approaches are disadvantaged by large power consumption, small communication bandwidth, and expensive equipment. Moreover, when the vehicle is operated near a seabed, the localization performance of the acoustic method is degraded by boundary reflections [14]. In contrast, optical methods offer high localization resolution and bandwidth, low energy consumption, and low price [15]. Consequently, they have been widely deployed in underwater close-range localization and communication in recent years [5,16]. Eren et al. [17] presented an optical detector array system that detects the precise pose of an unmanned underwater vehicle during its navigation. Bosch et al. [18,19] developed a method that tracks and localizes AUVs during close-range navigation. Their method relies on computer vision, active light markers, and an omnidirectional camera for fast and accurate pose estimation of the vehicle. Rust and Asada presented a dual-use visible light system that communicates with and localizes ROVs using modulated light signals [20]. However, these methods require light sources or several photosensitive sensors mounted on the underwater vehicle, which not only increase the energy consumption of the underwater submersible but also reduce the concealment and increase the cost.

This study was motivated by the use of hybrid underwater profilers (HUPs) in long-term persistent monitoring over a designated area [21,22]. The profilers need to transmit the collected oceanographic data to the shore base through a seafloor observatory network node and obtain accurate position information through that node. For long-term operation during missions (usually more than three months), the profiler must also minimize its energy use. In this study, a base station that connects to the seafloor observatory network and a retroreflector module that mounts on the profiler are designed, which are small-sized and cost efficient. Based on these designs, a laser-reflection approach is proposed for tracking, locating, and communicating with the underwater profiler. Underwater vehicles that use the proposed approach consume much less power and have a higher precision during localization than those that use conventional optical and acoustic methods.

**Figure 1:**
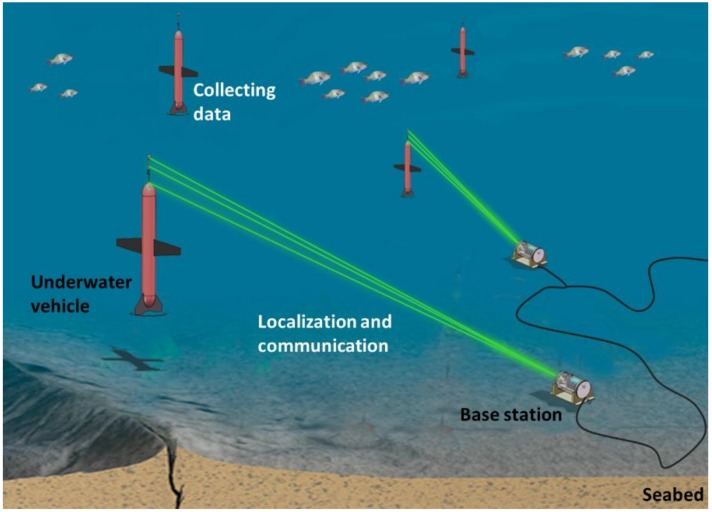
Illustration of the underwater vehicle localization and communication scheme based on laser reflection.

The workflow of this study about underwater profiler’s localization and communication is shown in Figure 1. The profiler first descends from the sea surface and collects oceanographic data. When it moves to near the seabed, it is tracked and located by the base station by means of laser reflection. The base station then sends the measured location and the task information to the profiler via optical communication, while the profiler sends the collected data to the base station and finally through the seafloor observatory network to the shore base.

The remainder of this paper is organized as follows: Section 2 describes the main components of the system and their corresponding coordinate systems. Section 3 presents the approaches for tracking, locating, recovering, and communicating with the profiler. Section 4 verifies the performance of the proposed approach in a series of experiments. Section 5 concludes the paper and suggests relevant future research.

## 2. System Description

A system that tracks, localizes, and communicates with the profiler comprises two main parts: a base station connected to the sea-network nodes, and a retroreflector module mounted on the profiler. The base station is equipped with a laser source and a charge-coupled device (CCD) camera, which detects the status of the tracking link and obtains the profiler’s position. The retroreflector module mounted on the profiler reflects the interrogating laser beam from the base station while communicating bi-directionally with the base station.

### 2.1. Base Station

**Figure 2:**
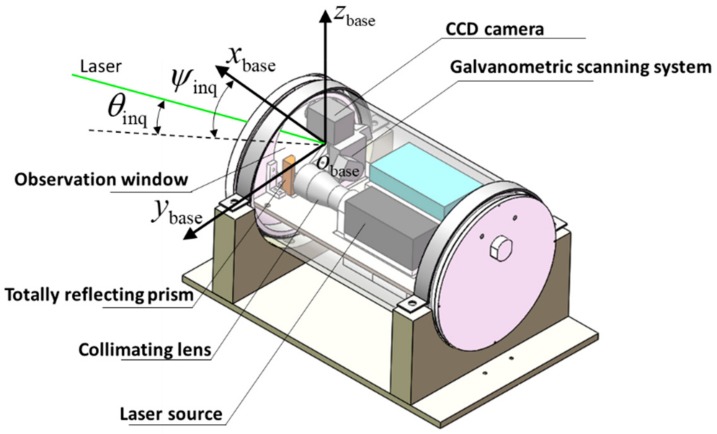
Major components and coordinate system of the base station.

As shown in Figure 2, the base station consists of three main parts: a laser source, a galvanometric scanning system, and a CCD camera. All components are encased in a watertight housing. The laser source outputs a laser beam with a wavelength of 532 nm, which is less attenuated in water than other wavelengths [23]. The laser light intensity is increased by a collimating lens placed in front of the laser source, which narrows the spread angle of the laser beam. The front of the collimating lens is a total reflecting prism that reflects the laser light into the galvanometric scanning system. The galvanometric scanning system comprises two rotatable mirrors driven by high-speed motors, which control the output direction (pitch and yaw) of the laser beam. The CCD camera receives and senses the laser reflected by the retroreflector module on the profiler.

Figure 2 also shows the coordinate system of the base station. The coordinate origin $O_{base}$ is set at the laser’s exit-point from the galvanometric scanning system. The x-axis $x_{base}$ coincides with the forward direction of the base station, while the y-axis $y_{base}$ points toward the left of the base station. The direction of the z-axis $z_{base}$ (upwards) is then determined via the right-hand rule. The emission angle of the laser from the base station $\Theta_{\mathrm{inq}}^{}=\;(\psi_{\mathrm{inq}},\theta_{\mathrm{inq}})$ comprises a yaw angle $\psi_{\mathrm{inq}}$ and a pitch angle $\theta_{\mathrm{inq}}$. The positive directions of $\psi_{\mathrm{inq}}$ and $\theta_{\mathrm{inq}}$ are defined as the clockwise direction along the $z_{base}$ and $y_{base}$ axes, respectively. Both angles are zero when the laser beam points toward $x_{base}$.

### 2.2. Retroreflector Module

**Figure 3:**
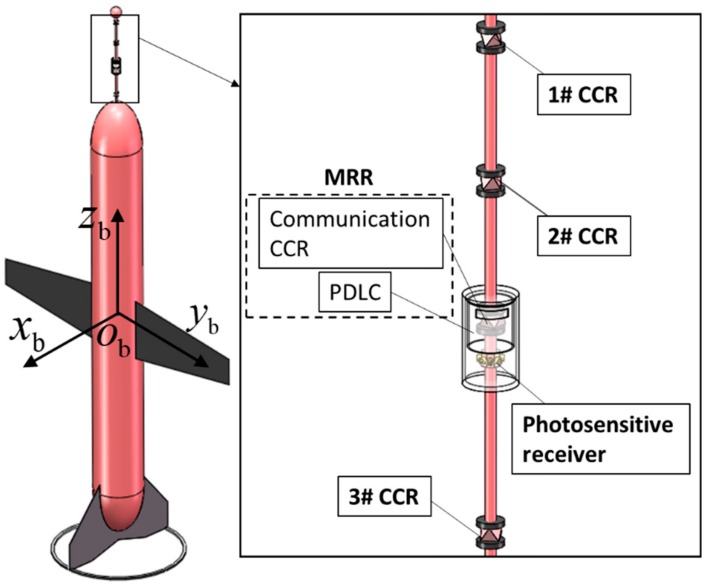
Major components and coordinate system of the retroreflector module mounted on the underwater profiler.

The retroreflector module is a detachable system mounted on the underwater profiler. The retroreflector module has three main functions: reflecting the laser interrogation beam, receiving the optical communication signal, and modulating the optical communication signal sent to the base station. Figure 3 shows the components of the retroreflector module—a corner cube retroreflector (CCR) array, a modulating retroreflector (MRR), and a photosensitive receiver—as well as the coordinate system of the retroreflector module on the underwater profiler. The origin of this system is the buoyancy center of the profiler $O_{b}$. The axes $z_{b}$ (longitudinal axis), $y_{b}$ and $x_{b}$ point to the head of the profiler, the right wing, and in the direction determined via the right-hand rule, respectively. The position and attitude states of the profiler are represented by $X_{\mathrm{prf}}=(P_{\mathrm{prf}},\Theta_{\mathrm{prf}})$. The attitude angle of the profiler is defined as $\Theta_{\mathrm{prf}}=(\psi_{\mathrm{prf}},\theta_{\mathrm{prf}})$, where $\psi_{\mathrm{prf}}$ and $\theta_{\mathrm{prf}}$ are the yaw and pitch angles of the profiler, respectively. The positive directions of $\psi_{\mathrm{prf}}$ and $\theta_{\mathrm{prf}}$ are the clockwise directions along the $x_{b}$ and $y_{b}$ axes, respectively. $P_{\mathrm{prf}}=(P_{\mathrm{prf},x},P_{\mathrm{prf},y},P_{\mathrm{prf},z})$ is the position of the profiler in the base-station coordinate system.

**Figure 4:**
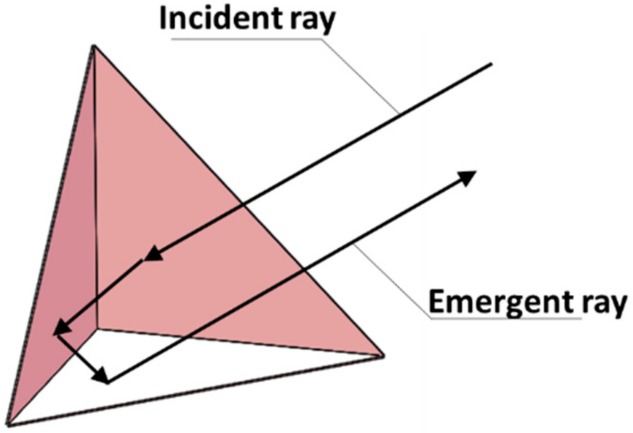
Optical path diagram of CCR.

The CCR is a passive object that ideally reflects the light to its source with minimal scattering and a 180° reflection angle (Figure 4). To enable tracking of the profiler in all directions, each CCR is omnidirectionally distributed around the $z_{b}$ axis. Tracking and localization is enabled by the retroreflector array with three CCRs that reflect the laser ray from the base station. The positions of CCRs 1#, 2#, and 3# in the profiler coordinate system are ***p***_r,1_ = (0, 0, *L*_r,1_), ***p***_r,2_ = (0, 0, *L*_r,2_), and $p_{r,3}=(0,\;0,\;L_{r,3})$, respectively. The MRR located at $p_{r,5}=(0,\;0,\;L_{r,5})$ in the profiler coordinate system communicates with the base station by modulating the reflected laser beam. The MMR contains two parts: a polymer-dispersed liquid crystal (PDLC) and a CCR. The PDLC is the optical modulator that controls the amount of light transmission. The optical receiver located at ***p***_r,4_ = (0, 0, *L*_r,4_) comprises six positive intrinsic-negative diodes around the $z_{b}$ axis, which receive the optical signal from the base station.

## 3. Approach

**Figure 5:**
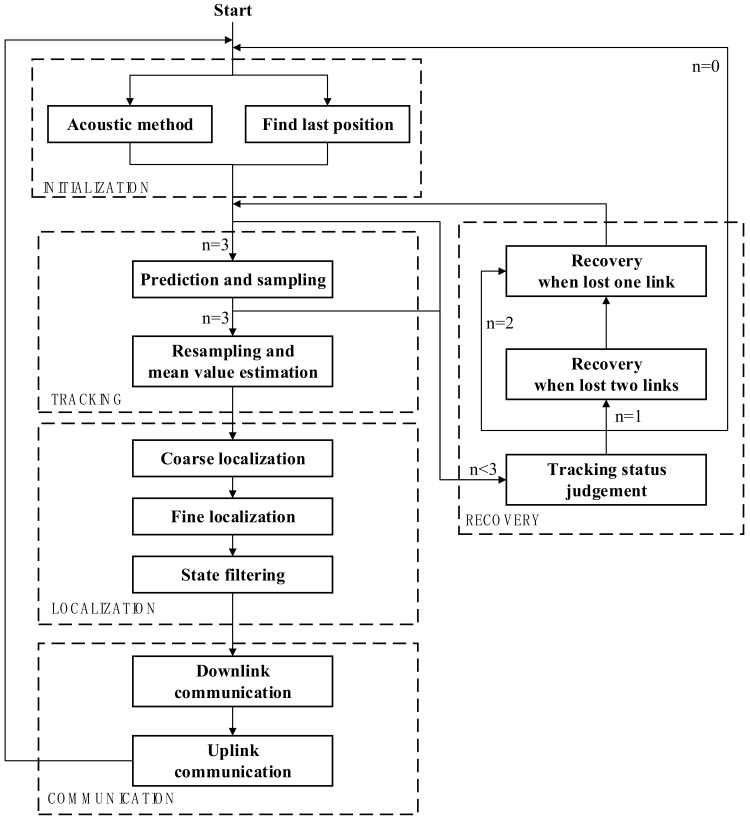
Workflow of the proposed approach.

Figure 5 shows the workflow of the proposed tracking, localization and communication approach, which is divided into five stages. In the initialization stage, the base station searches for the three CCRs in the most probable area of the profiler. Tracking begins when the base station establishes all laser-tracking links with the three CCRs on the profiler. The tracking stage is the basis of localization and communication stage in each work cycle, which consists of two steps: prediction and sampling, followed by resampling and mean-value estimation of the tracking angles. If any tracking link is lost in either step, the system enters in its recovery stage. After judging the tracking status, the system recovers the corresponding lost links. After completing the whole tracking cycle for all three links, the system enters the localization stage. The profile is coarsely located by the geometric relationship between the three tracking links, then finely located by optimizing the objective function using the least-squares method. At the end of the localization phase, the accuracy is improved by state filtering. Finally, the communication stage performs two-step (downlink and uplink) communications between the base station and the profiler. After completing the communication stage, the cycle restarts.

### 3.1. Initialization

The initialization stage determines the initial tracking angle of the base station. Depending on the state of the profiler, one of two initialization methods is selected. When the profiler descends from the sea surface and collects information near the seabed, the sea-network node locates the profiler by acoustics and sends the position information to the base station. Using this information, the base station calculates the initialization angle $\Theta_{\mathrm{inq},i,\mathrm{ini}}^{}=\;(\psi_{\mathrm{inq},i,\mathrm{ini}},\;\theta_{\mathrm{inq},i,\mathrm{ini}})\;$. Alternatively, when the profiler completes the monitoring near or on the seabed, the position of the profiler is almost unchanged. In this case, the position of the profiler at the end of the last cycle is assumed as the initial tracking position.

### 3.2. Tracking

**Figure 6:**
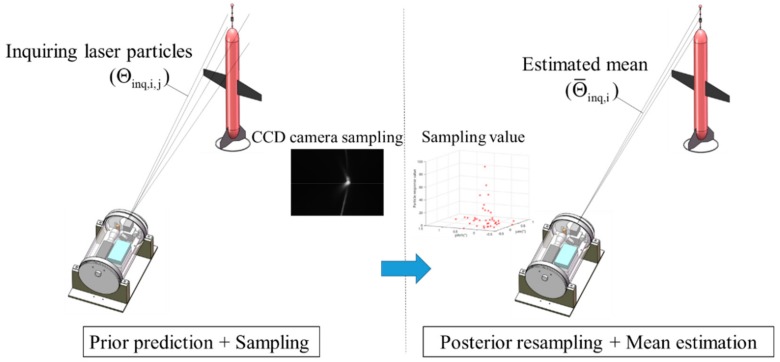
Schematic of the proposed tracking method.

The tracking stage maintains the laser-tracking link of each profiler CCR with the base station, ensuring that the laser beam can illuminate the corresponding CCR during the profiler’s movement. The proposed tracking method based on the particle filter involves two steps: prior prediction and sampling, and posterior resampling and mean estimation. Figure 6 is a schematic of the proposed tracking method. First, the laser beam state is predicted a priori from the state of the previous cycle, and the tracking object is sampled by a laser beam particle group constructed for that purpose. Based on the sampling response of each laser beam particle, the tracking object is resampled and the laser beam is estimated a posteriori from the important beam particles. The a priori prediction and a posteriori estimations are iterated to achieve continuous tracking of the CCR by the base station.

#### 3.2.1. Prediction and Sampling

The tracking angles during the tracking cycle are predicted from the angles of the previous cycle. The a priori tracking angle of the ith CCR of the j-th laser beam ${\overset{⌣}{\Theta }}_{\mathrm{inq},i,j,t}$ is calculated as:

(1) $${\overset{⌣}{\Theta }}_{\mathrm{inq},i,j,t}={\hat{\Theta }}_{\mathrm{inq},i,j,t-1}+\Delta \Theta_{\mathrm{inq},i,t-1}$$

where ${\hat{\Theta }}_{\mathrm{inq},i,j,t-1}$ is the posterior tracking angle of the ith CCR of the jth laser beam at time t−1, and $\Delta \Theta_{\mathrm{inq},i,t-1}=(\Delta \psi_{\mathrm{inq},i,t-1},\Delta \theta_{\mathrm{inq},i,t-1})$ represents the updated tracking angle between two adjacent cycles corresponding to the ith CCR. In the updated calculation, the profiler motion is assumed to have a constant velocity (CV), where:

(2) $$\left\{\begin{array}{c} \Delta \psi_{\mathrm{inq},i,t-1}=\psi_{\mathrm{inq},i,t-1}-\psi_{\mathrm{inq},i,t-2}\\ \Delta \theta_{\mathrm{inq},i,t-1}=\theta_{\mathrm{inq},i,t-1}-\theta_{\mathrm{inq},i,t-2} \end{array}\right.$$

After acquiring the a priori prediction angle of the laser beam, the laser beam direction is adjusted to a corresponding prediction angle ${\overset{⌣}{\Theta }}_{\mathrm{inq},i,j,t}$ for sampling the tracking object. There are $N_{\mathrm{tr}}$ laser beam particles in each tracking link.

**Figure 7:**
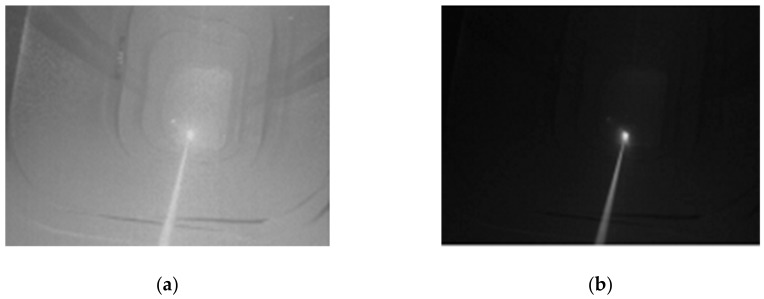
CCD camera imaging of the laser hitting a CCR. The exposure time of the CCD camera was set to 8400 μs in (**a**) and 500 μs in (**b**).

The CCD camera measures the retroreflective tracking signal a priori. When the retroreflective laser light intensity exceeds the CCD saturation voltage, the exposure time is reduced, and the ambient light can be filtered out, retaining only the laser-related pixels. The exposure time can be determined empirically. Panels (a) and (b) of Figure 7 show images of the laser hitting a CCR at exposure times of 8400 μs and 500 μs, respectively.

**Figure 8:**
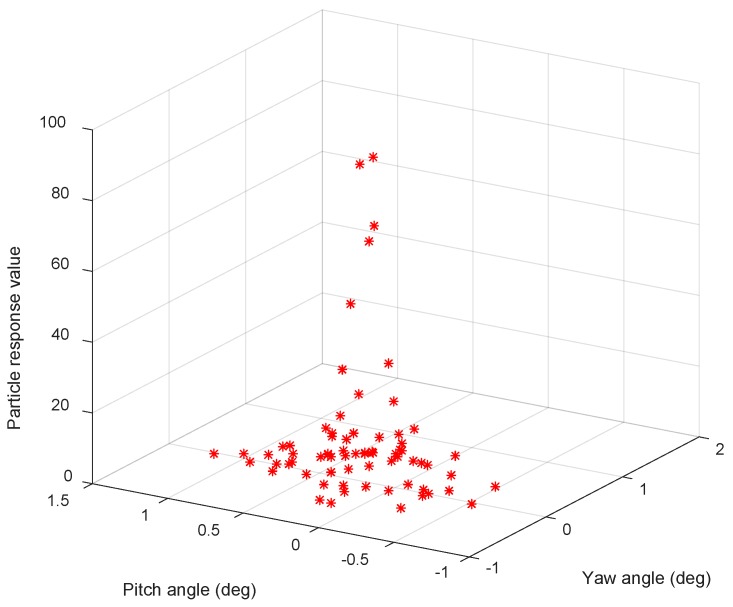
Base-station sampling responses of the ith CCR at time t.

In the tracking algorithm, the CCD response value $I_{\mathrm{res},i,j}$ defines the number of pixels in the image whose brightness exceeds the brightness threshold. Figure 8 shows the sampling responses of the tracking link of the ith CCR by the base-station laser at time t.

#### 3.2.2. Resampling and Mean Estimation

After the prediction and sampling processes, the resampling process inherits the particles with large importance and eliminates the trivial particles. When generating the a posteriori particles, the importance-resampling method takes the normalized importance of a particle as its resampled probability. The normalized sampling laser response is calculated as:

(3) $${\bar{I}}_{\mathrm{res},i,j}=I_{\mathrm{res},i,j}/\mathrm{sum}(I_{\mathrm{res},i})$$

where $\mathrm{sum}(I_{\mathrm{res},i})$ is the sum of the reflection responses of all laser beams corresponding to the ith CCR. As mentioned above, the a posteriori laser beam particle group ${\hat{\Theta }}_{\mathrm{inq},i,t}$ is generated by considering the sampled probability (assumed as the importance value) of each a priori laser beam. Importance resampling relies on the fact that the cumulative probability density of an arbitrary probability density conforms to the uniform distribution. Therefore, the importance of the particles transforms into the probability of being sampled. The resampling algorithm is given in Algorithm 1.


**Algorithm 1** Resampling algorithm1: ${\hat{\Theta }}_{\mathrm{inq},i,j,t}=\emptyset$, $S_{\mathrm{tr}}=0$
2: u is generated by the uniform probability of (0,1)3: **for**
*n* = 1 to $N_{\mathrm{tr}}$ do4: $S_{\mathrm{tr}}=S_{\mathrm{tr}}+{\bar{I}}_{\mathrm{res},i,n}$
5: **if**
$S_{\mathrm{tr}}>u$
6: ${\hat{\Theta }}_{\mathrm{inq},i,m,t}={\overset{⌣}{\Theta }}_{\mathrm{inq},i,n,t}$
7: **end if**8: **end for**9: **return**
${\hat{\Theta }}_{\mathrm{inq},i,j,t}$



The optimal tracking angle of the ith CCR ${\bar{\Theta }}_{\mathrm{inq},i,t}$ is estimated as:

(4) $${\bar{\Theta }}_{\mathrm{inq},i,t}=\frac{\sum_{j=1}^{N_{\mathrm{tr}}}{\hat{\Theta }}_{\mathrm{inq},i,j,t}}{N_{\mathrm{tr}}}$$

### 3.3. 3D Localization

After the tracking stage, the base station begins to locate the profiler based on the optimal estimated beam angle ${\bar{\Theta }}_{\mathrm{inq},i,t}$ of each tracking link in the tracking stage (Equation (4)) and the position of the CCR array in the profiler’s coordinate system. The localization proceeds by coarse localization, followed by fine localization.

#### 3.3.1. Coarse Localization

Coarse localization is based on the geometry of the base station and the profiler. Coarse localization assumes a vertical attitude of the profiler, which is approximately true when the profiler moves near the seabed. The coarse localization provides the distance between the base station and the profiler via the links of two CCRs. When three CCRs are available, three sources provide ranging information. Weighted averaging provides the rough distance from the profiler to the base station.

**Figure 9:**
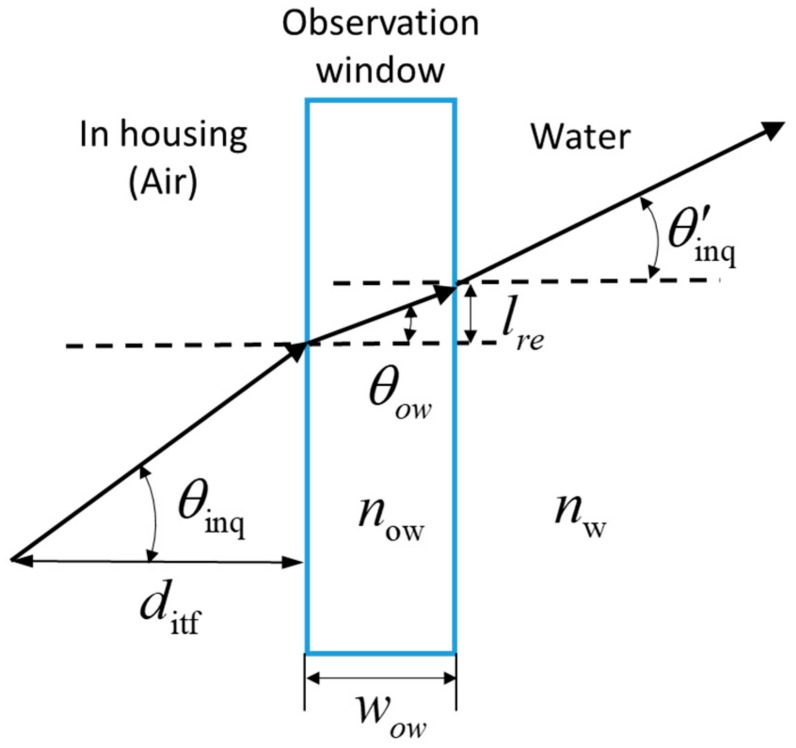
Light path of the laser emitted by the base station.

The localization stage must account for the refraction of the laser beam as it enters different media when passing from inside the base station through the window mirror into the water. As shown in Figure 9, a deviation $l_{re}$ is generated outside the mirror surface, the pitch angle of the laser beam also becomes ${\theta^{\prime }}_{\mathrm{inq}}^{}$. Using Snell’s law, the deviation $l_{re}$ is calculated as:

(5) $$l_{re}=w_{ow}\frac{\sin(\theta_{\mathrm{inq}})}{n_{ow}\sqrt{1-{\left(\frac{\sin(\theta_{\mathrm{inq}})}{n_{ow}}\right)}^{2}}}$$

where $\theta_{\mathrm{inq}}$ is the laser pitch angle in the air of the base station housing, and $n_{ow}$ and $w_{ow}$ are the refractive index and thickness of the observation window, respectively. As the observation window is very thin ($w_{ow}=2\;\mathrm{mm}$), the refractive deviation $l_{re}$ through the observation window can be ignored in practice. The pitch angles before and after the beam penetrates the water are related as follows:

(6) $$\frac{\sin(\theta_{\mathrm{inq}})}{\sin({\theta^{\prime }}_{\mathrm{inq}}^{})}=n_{w}$$

where ${\theta^{\prime }}_{\mathrm{inq}}^{}$ is the pitch angle after the laser beam enters the water, and $n_{w}$ is the refractive index of water.

**Figure 10:**
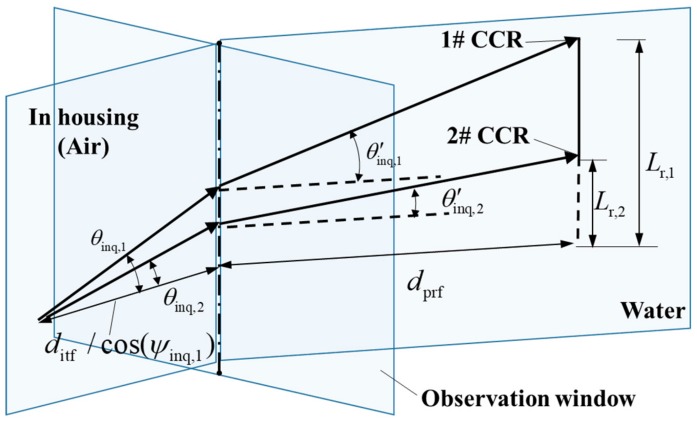
Schematic of the proposed triangulation ranging method, including the refraction effects.

Assuming a vertical pose of the profiler, this study proposes a triangulation ranging method that accounts for the refraction effects (see Figure 10). The distance from the profiler to the base station is computed as follows:

(7) $$\left\{\begin{array}{c} d_{\mathrm{prf}}=\frac{L_{r,1}-L_{r,2}-d_{\mathrm{itf}}/\cos(\psi_{\mathrm{inq},1}^{})(\tan(\theta_{\mathrm{inq},1})-\tan(\theta_{\mathrm{inq},2}))}{\left(\tan({\theta^{\prime }}_{\mathrm{inq},1}^{})-\tan({\theta^{\prime }}_{\mathrm{inq},2}^{})\right)}\\ {\theta^{\prime }}_{\mathrm{inq},1}^{}=\arcsin(\frac{\sin(\theta_{\mathrm{inq},1})}{n_{w}})\\ {\theta^{\prime }}_{\mathrm{inq},2}^{}=\arcsin(\frac{\sin(\theta_{\mathrm{inq},2})}{n_{w}}) \end{array}\right.$$

where $d_{\mathrm{itf}}$ is the distance from the origin of the profiler system to the base-station observation window. $\theta_{\mathrm{inq},1}$ and $\theta_{\mathrm{inq},2}$ are the pitch angles for tracking two CCRs in the base station’s housing, and ${\theta^{\prime }}_{\mathrm{inq},1}^{}$ and ${\theta^{\prime }}_{\mathrm{inq},2}^{}$ are the pitch angles of the corresponding laser beams in water. $p_{r,1}=(0,0,L_{r,1})$ and $p_{r,2}=(0,0,L_{r,2})$ are the origin positions of the two CCRs in the coordinate system of the profiler.

The distance of the profiler from the observation window of the base station is calculated as:

(8) $${\tilde{d}}_{\mathrm{prf}}=\frac{\sum_{i=1}^{3}d_{prf,i}}{3}$$

where $d_{prf,i}$ is estimated from the tracking angles of the ith pair of CCRs. $l_{\mathrm{lsr},i}$ is the line of the ith laser beam in water, described by a point $P_{\mathrm{lsr},i}=(P_{\mathrm{lsr},x,i},P_{\mathrm{lsr},y,i},P_{\mathrm{lsr},z,i})$ and a unit direction vector $\delta_{\mathrm{lsr},i}=(\delta_{\mathrm{lsr},x,i},\delta_{\mathrm{lsr},y,i},\delta_{\mathrm{lsr},z,i})$. The point $P_{\mathrm{lsr},i}$ is the intersection of the ith laser beam and the window mirror, and is explicitly given by:

(9) $$\{\begin{array}{c} P_{\mathrm{lsr},x,i}=d_{\mathrm{itf}}\\ P_{\mathrm{lsr},y,i}=d_{\mathrm{itf}}\tan(\psi_{\mathrm{inq},i}^{})\\ P_{\mathrm{lsr},z,i}=d_{\mathrm{itf}}\tan(\theta_{\mathrm{inq},i}^{})/\cos(\psi_{\mathrm{inq},i}^{})\\ \left(\delta_{\mathrm{lsr},x,i},\delta_{\mathrm{lsr},y,i},\delta_{\mathrm{lsr},z,i}\right)=\left(T_{refr,3,1},T_{refr,3,2},T_{refr,3,3}\right)\\ T_{refr}=\left[\begin{array}{cccc} \cos(\psi_{\mathrm{inq},i}^{})\cos(\theta_{\mathrm{inq},i}^{}) & -\sin(\psi_{\mathrm{inq},i}^{}) & \cos(\psi_{\mathrm{inq},i}^{})\sin(\theta_{\mathrm{inq},i}^{}) & 0\\ \sin(\psi_{\mathrm{inq},i}^{})\cos(\theta_{\mathrm{inq},i}^{}) & \cos(\psi_{\mathrm{inq},i}^{}) & \sin(\psi_{\mathrm{inq},i}^{})\sin(\theta_{\mathrm{inq},i}^{}) & 0\\ -\sin(\theta_{\mathrm{inq},i}^{}) & 0 & \cos(\theta_{\mathrm{inq},i}^{}) & 0\\ 0 & 0 & 0 & 1 \end{array}\right]T_{2}\\ T_{2}=\left[\begin{array}{cccc} 1-2(q_{2}^{2}+q_{3}^{2}) & 2(q_{1}q_{2}-q_{0}q_{3}) & 2(q_{0}q_{2}+q_{1}q_{3}) & 0\\ 2(q_{1}q_{2}+q_{0}q_{3}) & 1-2(q_{1}^{2}+q_{3}^{2}) & 2(q_{2}q_{3}-q_{0}q_{1}) & 0\\ 2(q_{1}q_{3}-q_{0}q_{2}) & 2(q_{0}q_{1}+q_{2}q_{3}) & 1-2(q_{1}^{2}+q_{2}^{2}) & 0\\ 0 & 0 & 0 & 1 \end{array}\right]\\ q=(q_{0},q_{1},q_{2},q_{3}) \end{array}$$

where $T_{refr}$ represents the rotation matrix of the laser beam from its initial position to its final position in the water, and $T_{2}$ denotes the rotation matrix of the laser beam after refraction at the window mirror. The quaternion q transforms the laser from the air to the water through the rotation matrix, and is calculated as follows:

(10) $$q=\left(\cos(\varphi_{\mathrm{refr}}/2),n_{\mathrm{refr}}\sin(\varphi_{\mathrm{refr}}/2)\right)$$

where $\varphi_{\mathrm{refr}}$ is the deflection angle of the laser beam from air to water, and $n_{\mathrm{refr}}$ is the unit vector along the deflection axis, expressed as:

(11) $$\left\{\begin{array}{c} n_{\mathrm{refr}}=\nu_{1}\times \nu_{2}/(\left\|\nu_{1}\right\|\cdot \left\|\nu_{2}\right\|)\\ \nu_{1}=(1,\tan(\psi_{\mathrm{inq},i}^{}),\tan(\theta_{\mathrm{inq},i}^{})/\cos(\psi_{\mathrm{inq},i}^{}))\\ \nu_{2}=(1,0,0)\\ \varphi_{\mathrm{refr}}=\beta_{0}-\arcsin(\sin(\beta_{0})/n_{w})\\ \beta_{0}=\arcsin(\left\|\nu_{1}\times \nu_{2}\right\|) \end{array}\right.$$

In (11), $\nu_{1}$ is the direction vector of the laser beam before refraction and $\nu_{2}$ is the normal vector of the observation window. Therefore, the profiler is coarsely located at ${\tilde{P}}_{\mathrm{prf}}=({\tilde{P}}_{\mathrm{prf},x},{\tilde{P}}_{\mathrm{prf},y},{\tilde{P}}_{\mathrm{prf},z})$ with:

(12) $$\left\{\begin{array}{c} {\tilde{P}}_{\mathrm{prf},x}={\tilde{d}}_{\mathrm{prf}}\delta_{\mathrm{lsr},x,1}/\sqrt{1-\delta^{2}{}_{\mathrm{lsr},z,1}}+P_{\mathrm{lsr},x,1}\\ {\tilde{P}}_{\mathrm{prf},y}={\tilde{d}}_{\mathrm{prf}}\delta_{\mathrm{lsr},y,1}/\sqrt{1-\delta^{2}{}_{\mathrm{lsr},z,1}}+P_{\mathrm{lsr},y,1}\\ {\tilde{P}}_{\mathrm{prf},z}={\tilde{d}}_{\mathrm{prf}}\delta_{\mathrm{lsr},z,1}/\sqrt{1-\delta^{2}{}_{\mathrm{lsr},z,1}}+P_{\mathrm{lsr},z,1}-L_{r,1} \end{array}\right.$$

#### 3.3.2. Fine Localization

The three tracking links are sequentially sampled in one computing cycle, meaning that the optimal estimated tracking angle ${\bar{\Theta }}_{\mathrm{inq},i}$ obtained by the earlier sampling link lags that of the later sampling link. As this lag affects the subsequent optimization calculation, we must synchronize the first two tracking links (${\bar{\Theta }}_{\mathrm{inq},1}$ and ${\bar{\Theta }}_{\mathrm{inq},2}$) with the last tracking link ${\bar{\Theta }}_{\mathrm{inq},3}$. Assuming that the laser beam angles of each tracking link vary with uniform velocity in the recently calculated cycles, and taking the sampling time of the third tracking link as the time baseline, the angle of the ith tracking link after synchronization is ${\bar{\Theta }}_{\mathrm{inq},i,t}^{*}=({\bar{\psi }}_{\mathrm{inq},i,t}^{*},{\bar{\theta }}_{\mathrm{inq},i,t}^{*})$. The components are computed as follows:

(13) $$\left\{\begin{array}{c} {\bar{\psi }}_{\mathrm{inq},i,t}^{*}={\bar{\psi }}_{\mathrm{inq},3,t}^{}+\frac{3-i}{3}({\bar{\psi }}_{\mathrm{inq},i,t}^{}-{\bar{\psi }}_{\mathrm{inq},i,t-1}^{})\\ {\bar{\theta }}_{\mathrm{inq},i,t}^{*}={\bar{\theta }}_{\mathrm{inq},3,t}^{}+\frac{3-i}{3}({\bar{\theta }}_{\mathrm{inq},i,t}^{}-{\bar{\theta }}_{\mathrm{inq},i,t-1}^{}) \end{array}\right.$$

The synchronization correction of the tracking links ensures that the tracking angle errors of the links are approximately Gaussian distributed about the same time baseline. This condition is prerequisite for fine localization.

**Figure 11:**
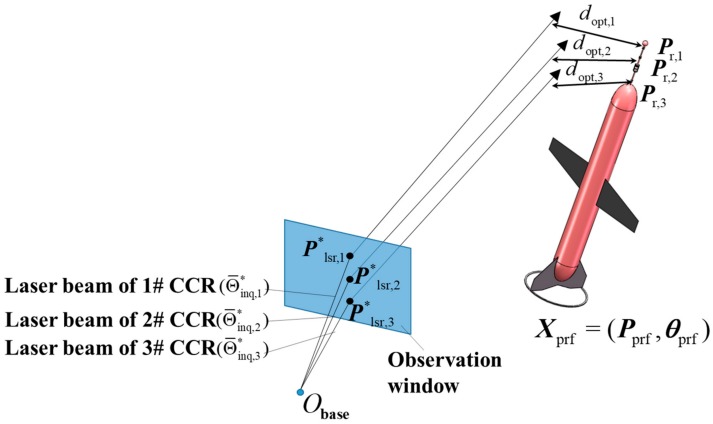
Illustration of the proposed fine localization.

The profiler location is optimized by the least-squares method. The laser beam is divided into a part in the base station housing (air) and a part in the water (Figure 11). The core problem is to minimize the squares of the distances $d_{\mathrm{opt},i}$ from the CCRs to their corresponding tracking laser beams. The position of the ith CCR in the base-station coordinate system, defined as:

(14) $$P_{r,i}=(P_{r,x,i},P_{r,y,i},P_{r,z,i})$$

can be calculated as:

(15) $${\left(P_{r,i}^{},1\right)}^{T}=T_{\mathrm{prf}}{\left(p_{r,i}^{},1\right)}^{T}$$

where $p_{r,i}^{}=(p_{r,x,i}^{},p_{r,y,i}^{},p_{r,z,i}^{})$ is the position of the ith CCR in the profiler coordinate system. The transformation matrix $T_{\mathrm{prf}}$ between the coordinate systems of the profiler and the base station is given as follows:

(16) $$T_{\mathrm{prf}}=\left[\begin{array}{cccc} \cos(\psi_{\mathrm{prf}})\cos(\theta_{\mathrm{prf}}) & -\sin(\psi_{\mathrm{prf}}) & \cos(\psi_{\mathrm{prf}})\sin(\theta_{\mathrm{prf}}) & P_{\mathrm{prf},x}\\ \sin(\psi_{\mathrm{prf}})\cos(\theta_{\mathrm{prf}}) & \cos(\psi_{\mathrm{prf}}) & \sin(\psi_{\mathrm{prf}})\sin(\theta_{\mathrm{prf}}) & P_{\mathrm{prf},y}\\ -\sin(\theta_{\mathrm{prf}}) & 0 & \cos(\theta_{\mathrm{prf}}) & P_{\mathrm{prf},z}\\ 0 & 0 & 0 & 1 \end{array}\right]$$

The laser beam in the water $l^{*}{}_{\mathrm{lsr},i}$ is described by a point $P^{*}{}_{\mathrm{lsr},i}$ on the window mirror and a unit direction vector $\delta^{*}{}_{\mathrm{lsr},i}$, where:

(17) $$\left\{\begin{array}{l} P^{*}{}_{\mathrm{lsr},i}=(P^{*}{}_{\mathrm{lsr},x,i},P^{*}{}_{\mathrm{lsr},y,i},P^{*}{}_{\mathrm{lsr},z,i})\\ \delta^{*}{}_{\mathrm{lsr},i}=(\delta^{*}{}_{\mathrm{lsr},x,i},\delta^{*}{}_{\mathrm{lsr},y,i},\delta^{*}{}_{\mathrm{lsr},z,i}) \end{array}\right.$$

The distance from the ith CCR $P_{r,i}$ to its tracking beam $l^{*}{}_{\mathrm{lsr},i}$ is then calculated as:

(18) $$d_{\mathrm{opt},i}=\frac{\left\|\varepsilon_{\mathrm{tmp},i}\times \delta^{*}{}_{\mathrm{lsr},i}\right\|}{\left\|\delta^{*}{}_{\mathrm{lsr},i}\right\|}$$

where $\varepsilon_{\mathrm{tmp},i}$ is the vector formed by $P^{*}{}_{\mathrm{lsr},i}$ and $P_{r,i}$:

(19) $$\varepsilon_{\mathrm{tmp},i}=P_{r,i}-P^{*}{}_{\mathrm{lsr},i}$$

The objective function based on the least-squares method is defined as the sum of the squared distances between the CCRs and the paths of their corresponding laser beams (Figure 11):

(20) $$cost(X_{\mathrm{prf}})=\sum_{i=1}^{3}d_{\mathrm{opt},i}^{2}$$

The optimal position of the profiler $X_{\mathrm{prf}}^{*}=(P_{\mathrm{prf}}^{*},\Theta_{\mathrm{prf}}^{*})$ is then obtained as:

(21) $$X_{\mathrm{prf}}^{*}=\underset{X_{\mathrm{prf}}}{\arg\min}cost(X_{\mathrm{prf}})$$

Note that the minimization in (21) is a nonlinear optimization problem that can be solved by the Broyden–Fletcher–Goldfarb–Shanno (BFGS) algorithm of the quasi-Newton method [24,25]. The BFGS algorithm executes with high efficiency and without needing to calculate a precise Hessian matrix. The initial position of the quasi-Newton iteration is the optimal position found in the previous cycle.

#### 3.3.3. State Filtering

To reduce the influence of noise on the localization results, the profiler state is estimated by the Kalman filter (KF) method.

##### State and Observation Model

When the profiler moves near the seabed, its speed is small and almost constant; thus, its motion model approximates a CV model. The state model is expressed as:

(22) $$S_{\mathrm{prf},t}=A_{f}S_{\mathrm{prf},t-1}^{}+w_{t}$$

where $S_{\mathrm{prf},t}$ is the state vector of the profiler at time t:

(23) $$S_{\mathrm{prf},t}={\left[P_{\mathrm{prf},x,t}\;P_{\mathrm{prf},y,t}\;P_{\mathrm{prf},z,t}\;V_{\mathrm{prf},x,t}\;V_{\mathrm{prf},y,t}\;V_{\mathrm{prf},z,t}\right]}^{T}$$

In (23), $P_{\mathrm{prf},x}\;P_{\mathrm{prf},y}$ and $P_{\mathrm{prf},z}$ are the positions of the profiler and $V_{\mathrm{prf},x}\;V_{\mathrm{prf},y}$ and $V_{\mathrm{prf},z}$ are their corresponding velocities. In Equation (22), the transformation matrix $A_{f}$ is calculated as:

(24) $$A_{f}=\left[\begin{array}{cccccc} 1 & 0 & 0 & \Delta t & 0 & 0\\ 0 & 1 & 0 & 0 & \Delta t & 0\\ 0 & 0 & 1 & 0 & 0 & \Delta t\\ 0 & 0 & 0 & 1 & 0 & 0\\ 0 & 0 & 0 & 0 & 1 & 0\\ 0 & 0 & 0 & 0 & 0 & 1 \end{array}\right]$$

and $w_{t}$ is the process noise, which is a Gaussian white noise with zero mean and variance $Q_{f}$. The observation model can be expressed as:

(25) $$Z_{\mathrm{prf},t}=H_{f}S_{\mathrm{prf},t-1}^{}+v_{t}$$

where $Z_{\mathrm{prf},t}$ is the observation value at time t, which is actually the profiler position estimated by the fine localization method in Section 3.3.2. It is obtained as:

(26) $$Z_{\mathrm{prf},t}=P_{\mathrm{prf},t}^{*}$$

The measurement noise $v_{t}$ is also a Gaussian white noise with zero mean and variance $R_{f}$. The measurement matrix $H_{f}$ in (25) is given by:

(27) $$H_{f}=\left[\begin{array}{cccccc} 1 & 0 & 0 & 0 & 0 & 0\\ 0 & 1 & 0 & 0 & 0 & 0\\ 0 & 0 & 1 & 0 & 0 & 0 \end{array}\right]$$

##### Prediction

The predicted state ${\hat{S}}_{\mathrm{prf},t}$ and its associated covariance matrix ${\bar{P}}_{f,t}$ are respectively given by:

(28) $${\hat{S}}_{\mathrm{prf},t}=A_{f}S_{\mathrm{prf},t-1}^{*}$$

(29) $${\bar{P}}_{f,t}=A_{f}P_{f,t-1}A_{f}^{T}+Q_{f}$$

where $S_{\mathrm{prf},t-1}^{*}$ is the optimally estimated state of the profiler at time t−1. The covariance matrix of the process noise $Q_{f}$ is:

(30) $$Q_{f}=\left[\begin{array}{cccccc} 0 & 0 & 0 & 0 & 0 & 0\\ 0 & 0 & 0 & 0 & 0 & 0\\ 0 & 0 & 0 & 0 & 0 & 0\\ 0 & 0 & 0 & \sigma_{\mathrm{vx}}^{2} & 0 & 0\\ 0 & 0 & 0 & 0 & \sigma_{\mathrm{vy}}^{2} & 0\\ 0 & 0 & 0 & 0 & 0 & \sigma_{\mathrm{vx}}^{2} \end{array}\right]$$

where $\sigma_{\mathrm{vx}}^{2}$, $\sigma_{\mathrm{vy}}^{2}$ and $\sigma_{\mathrm{vz}}^{2}$ represent the variances of the profiler velocities in the $x_{\mathrm{base}}$, $y_{\mathrm{base}}$ and $z_{\mathrm{base}}$ directions, respectively, in adjacent calculation cycles.

##### Update

The Kalman gain $K_{f,t}$ is computed as:

(31) $$K_{f,t}={\bar{P}}_{f,t}H_{f}^{T}{\left(H_{f}{\bar{P}}_{f,t}H_{f}^{T}+R_{f}\right)}^{-1}$$

Combining the predicted state and the measured position of the profiler, the optimally estimated state vector of the profiler $S_{\mathrm{prf},t}^{*}$ and its covariance matrix $P_{f,t}$ at time t are calculated as:

(32) $$S_{\mathrm{prf},t}^{*}={\hat{S}}_{\mathrm{prf},t}+K_{f,t}(Z_{\mathrm{prf},t}-H_{f}{\hat{S}}_{\mathrm{prf},t})$$

(33) $$P_{f,t}=(I-K_{f,t}H_{f}){\bar{P}}_{f,t}$$

where the covariance matrix of the measurement noise $R_{f}$ is given by:

(34) $$R_{f}=\left[\begin{array}{ccc} \sigma_{x}^{2} & 0 & 0\\ 0 & \sigma_{y}^{2} & 0\\ 0 & 0 & \sigma_{z}^{2} \end{array}\right]$$

**Figure 12:**
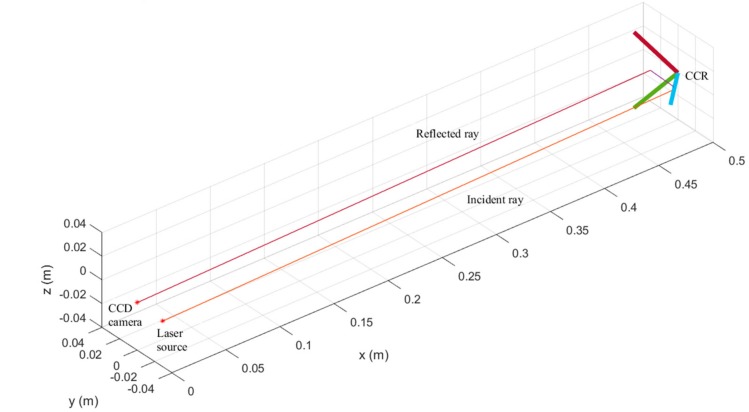
Simulation scenario of tracking and localization by the proposed method, showing the optical path.

In (34), $\sigma_{x}^{2}$, $\sigma_{y}^{2}$, and $\sigma_{z}^{2}$ represent the squared errors in the three-dimensional position of the profiler, obtained via the least-squares method. The proposed tracking and localization algorithm was verified via a MATLAB simulation. The simulation setup comprised the laser source, the laser beams, a CCD camera model, and a CCR module. The simulated tracking and localization situation is visualized in Figure 12. The thick solid lines with three different colors represent the three edges of a CCR, which form a simplified representation of a CCR. The optical path during localization and tracking can be clearly observed via simulation.

**Figure 13:**
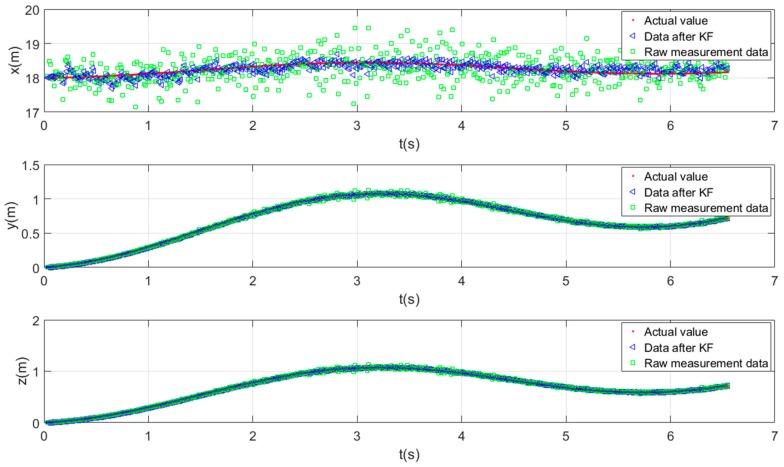
Simulated localization results as functions of time.

In the simulation, the underwater profiler performs sinusoidal motion on the $x_{\mathrm{base}}$, $y_{\mathrm{base}}$, and $z_{\mathrm{base}}$ axes. The base station uses the proposed tracking and localization approach to locate the profiler. Figure 13 shows the time-varying positions of the profiler along the three axes in the base-station coordinate system, $x_{\mathrm{base}}$, $y_{\mathrm{base}}$, and $z_{\mathrm{base}}$ (top to bottom). Plotted are the actual positions and the results of applying and omitting the KF.

The tracking errors of the proposed method with and without KF are given in Table 1. The accuracy criterion was the root mean squared error (RMSE). After KF, the RMSEs in the $x_{\mathrm{base}}$, $y_{\mathrm{base}}$, and $z_{\mathrm{base}}$ coordinates were reduced by 69%, 57%, and 57%, respectively, relative to the unfiltered data. The results confirm that the KF significantly improved the localization accuracy.

### 3.4. Recovery

During the actual tracking process, the tracking link to the CCR may be lost. The base station needs to recover any lost links by operating the tracking recovery algorithm. The recovery algorithm proceeds in two steps: tracking the status judgment and searching for missing CCRs.

#### 3.4.1. Tracking Status Judgment

The status of the tracking links is usually obtained by setting a fixed threshold value. The maximum signal strength $I_{\mathrm{res},i,\max}$ of the CCD camera receiving the reflected laser beam is compared with the threshold $\lambda_{\mathrm{thr}}$. If the signal strength exceeds the threshold (i.e., $I_{\mathrm{res},i,\max}>\lambda_{\mathrm{thr}}$), the state is considered as valid; otherwise it is invalid.

The fixed threshold method is simple but defective in one aspect. As the profiler gradually moves away from the base station, the signal strength received by the CCD camera $I_{\mathrm{res},i,\max}$ gradually decreases. Therefore, if the signal strength falls below the threshold ($I_{\mathrm{res},i,\max}<\lambda_{\mathrm{thr}}$), a valid tracking state will be misjudged as a tracking failure.

To dynamically adjust the threshold $\lambda_{\mathrm{thr}}$ and reduce the misjudgment probability, we update $\lambda_{\mathrm{thr}}$ by a tracking status judgment method based on K-means clustering [26], and then determine the tracking state. K-means clustering divides the signal strengths of the CCD camera into k categories. In the present case there were two clusters (k=2): a cluster of signal strengths with invalid tracking links (cluster $C_{1}$), and a cluster of signal strengths with valid tracking links (cluster $C_{2}$). $I_{p}$ is the signal strength collected during the tracking process. To increase the judgment accuracy, two samples were collected in each period, one before the beginning of the tracking period ($I_{2\nu -1}$, n = 1, 2, …), and another during tracking ($I_{2\nu }$, n = 1, 2, …). Let $\mu_{1}$ and $\mu_{2}$ be the centroids of clusters $C_{1}$ and $C_{2}$, respectively. The signal strengths in the first period $I_{1}$ (before the beginning of each tracking) and $I_{2}$ (during tracking with valid status) were selected as the initial centroids of clusters $C_{1}$ and $C_{2}$, respectively. During the tracking, each new signal strength was assigned to the cluster whose mean minimized the squared Euclidean distance between itself and the signal:

(35) $$C_{i}=\{I_{p}:{\left\|I_{p}-\mu_{i}\right\|}^{2}\leq {\left\|I_{p}-\mu_{j}\right\|}^{2}\forall j,1\leq j\leq k\}$$

where each $I_{p}$ was assigned to $C_{i}$. The centroids were updated by calculating the means of the clusters:

(36) $$\mu_{i}=\frac{\sum_{I_{j}\in C_{i}}I_{j}}{\left|C_{i}\right|}$$

If the new signal strength collected at the time of tracking ($I_{2\nu }$) is assigned to cluster $C_{1}$, then the tracking status is judged as invalid and the tracking recovery algorithm is invoked.

#### 3.4.2. Tracking Recovery Algorithm

The core task of the tracking recovery algorithm is searching the missing tracking links. Different procedures are invoked for one or two lost tracking links.

##### Loss of One Tracking Link

**Figure 14:**
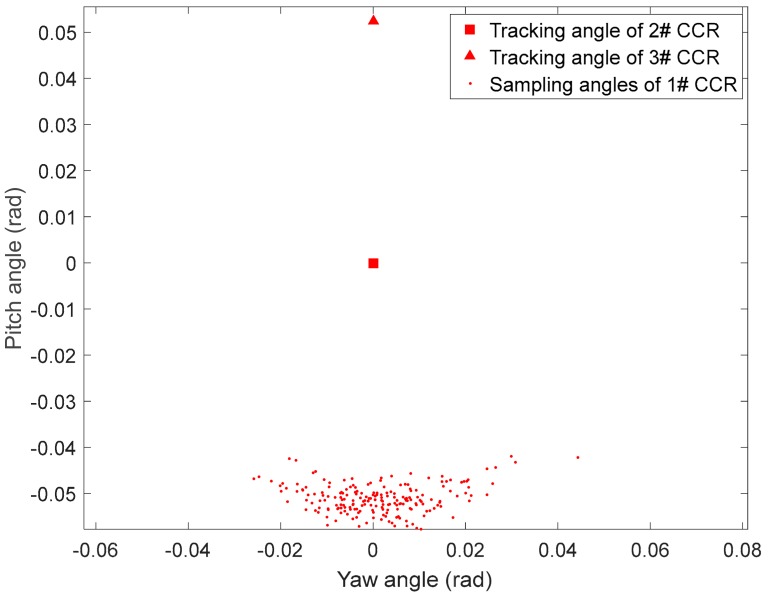
Recovery sampling angles after losing 1# CCR.

When two tracking links are valid, the angle of the corresponding interrogation laser $\Theta_{\mathrm{inq},i,t}$ of the failed link is estimated by linear interpolation. Suppose (for example) that the tracking link corresponding to 1# CCR is lost, and the links corresponding to 2# and 3# CCRs are valid. The sampling angle of the laser beam to the 1# CCR angle ${\tilde{\Theta }}_{\mathrm{inq},1,t}^{}$ can be calculated as:

(37) $$\left\{\begin{array}{c} {\tilde{\Theta }}_{\mathrm{inq},1,t}^{T}=\Theta_{\mathrm{inq},2,t}^{T}+\left[\begin{array}{cc} \cos(\theta_{\xi }) & -\sin(\theta_{\xi })\\ \sin(\theta_{\xi }) & \cos(\theta_{\xi }) \end{array}\right]\left[\begin{array}{c} \psi_{\mathrm{inq},2,t}-\psi_{\mathrm{inq},3,t}\\ \theta_{\mathrm{inq},2,t}-\theta_{\mathrm{inq},3,t} \end{array}\right]\frac{L_{r,1}-L_{r,2}}{L_{r,2}-L_{r,3}}(1+\lambda_{\xi })\\ \theta_{\xi }\sim N(0,\sigma_{\theta_{\xi }}^{2})\\ \lambda_{\xi }\sim N(0,\sigma_{\lambda_{\xi }}^{2}) \end{array}\right.$$

where $\Theta_{\mathrm{inq},2,t}^{}=(\psi_{\mathrm{inq},2,t},\theta_{\mathrm{inq},2,t})$ and $\Theta_{\mathrm{inq},3,t}^{}=(\psi_{\mathrm{inq},3,t},\theta_{\mathrm{inq},3,t})$ are the angles of the tracking links corresponding to 2# and 3# CCRs, respectively. $\theta_{\xi }$ and $\lambda_{\xi }$ are the angle and length uncertainties in $\Theta_{\mathrm{inq},1,t}$, respectively, which follow Gaussian distributions with zero mean and variances of $\sigma_{\theta_{\xi }}^{2}$ and $\sigma_{\lambda_{\xi }}^{2}$, respectively. Figure 14 shows the recovery search angle when the corresponding link to 1# CCR is lost. The corresponding parameters are $L_{r,1}=0.15$, $L_{r,2}=0$, $L_{r,3}=-0.15$, $\Theta_{\mathrm{inq},2,t}^{}=(0,0)$, $\Theta_{\mathrm{inq},3,t}^{}=(0,0.0523)$, $\theta_{\xi }\sim N(0,0.188)$, $\lambda_{\xi }\sim N(0,0.06)$. As shown in the figure, the search area was fan-shaped, and the search for the missing CRR was intensified in regions of dense search points.

##### Loss of Two Tracking Links

**Figure 15:**
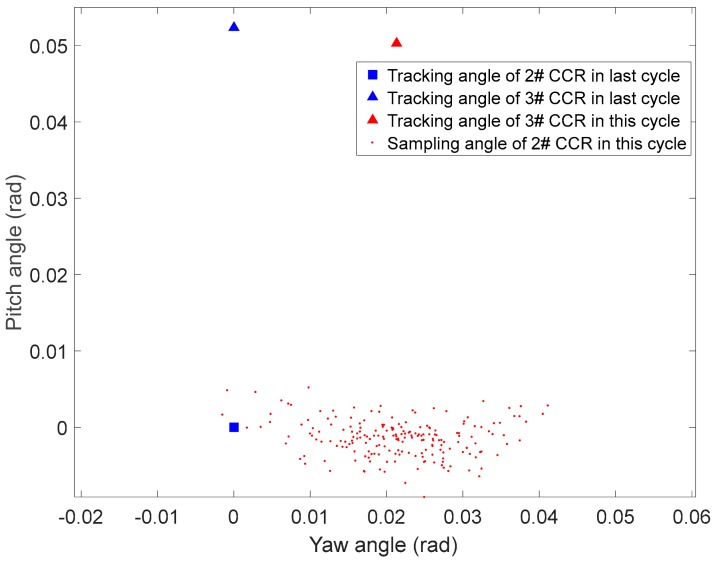
Recovery sampling angles when 1# and 2# CCRs are lost.

When two tracking links are lost, the algorithm first recovers one tracking link, and then retrieves the last link by the previously described method. Suppose (for example) that the tracking links corresponding to 1# and 2# CCRs are lost, but the link corresponding to 3# CCR is valid. The link corresponding to 2# CCR needs to be restored first. Using the tracking angles of the previous and current cycles, the sampling angle ${\tilde{\Theta }}_{\mathrm{inq},2,t}^{}$ is generated as follows:

(38) $$\left\{\begin{array}{c} {\tilde{\Theta }}_{\mathrm{inq},2,t}^{T}=\Theta_{\mathrm{inq},3,t}^{T}+\left[\begin{array}{cc} \cos(\theta_{\xi }) & -\sin(\theta_{\xi })\\ \sin(\theta_{\xi }) & \cos(\theta_{\xi }) \end{array}\right]\left[\begin{array}{c} \psi_{\mathrm{inq},2,t-1}-\psi_{\mathrm{inq},3,t-1}\\ \theta_{\mathrm{inq},2,t-1}-\theta_{\mathrm{inq},3,t-1} \end{array}\right](1+{\lambda^{\prime }}_{\xi }^{})\\ {\theta^{\prime }}_{\xi }^{}\sim N(0,\sigma_{\theta_{\xi }^{’}}^{2’})\\ {\lambda^{\prime }}_{\xi }^{}\sim N(0,\sigma_{\lambda_{\xi }^{’}}^{2’}) \end{array}\right.$$

where $\Theta_{\mathrm{inq},2,t-1}^{}=(\psi_{\mathrm{inq},2,t-1},\theta_{\mathrm{inq},2,t-1})$ and $\Theta_{\mathrm{inq},3,t-1}^{}=(\psi_{\mathrm{inq},3,t-1},\theta_{\mathrm{inq},3,t-1})$ are the tracking angles corresponding to 2# and 3# CCRs, respectively, in the previous cycle, and $\Theta_{\mathrm{inq},3,t}^{}=(\psi_{\mathrm{inq},3,t},\theta_{\mathrm{inq},3,t})$ is the tracking angle corresponding to 3# CCR in the current cycle. ${\theta^{\prime }}_{\xi }^{}$ and ${\lambda^{\prime }}_{\xi }^{}$ are the angle and length uncertainties of $\Theta_{\mathrm{inq},2,t}$, respectively, which follow Gaussian distributions with zero mean and variances of $\sigma_{\theta_{\xi }^{’}}^{2’}$ and $\sigma_{\lambda_{\xi }^{’}}^{2’}$, respectively. Figure 15 shows the recovery sampling angles of 2# CRR when links 1# and 2# are lost. The corresponding parameters are $\Theta_{\mathrm{inq},2,t-1}^{}=(0,0)$, $\Theta_{\mathrm{inq},3,t-1}^{}=(0,0.0523)$, $\Theta_{\mathrm{inq},3,t}^{}=(0.0213\;\mathrm{rad},0.0503\;\mathrm{rad})$, ${\theta^{\prime }}_{\xi }\sim N(0,0.188)$, and ${\lambda^{\prime }}_{\xi }\sim N(0,0.06)$.

After recovering one tracking link, the remaining link can be recovered via the one-link-lost recovery algorithm as described before.

### 3.5. Communication

For transmitting the oceanography data collected by the profiler to the base station, we propose a bidirectional communication approach, wherein the profiler also receives the command sent by the base station. To reduce the power consumption of profiler monitoring, the profiler and the base station communicate via a laser-reflection modulation method. The direction of information transmission is divided into downlink (base station to profiler) and uplink (profiler to base station) transmission.

#### 3.5.1. Downlink

As described in Section 2.2, the optical information is received by an omnidirectional photosensitive receiver mounted at position $p_{r,4}=(0,0,L_{r,4})$ of the profiler in the body coordinate system. In downlink transmission, the coded laser from the base station illuminates the photosensitive receiver on the profiler, and the data are transmitted while tracking the three CCRs. Downlink communication proceeds in two steps: laser beam alignment and laser coding.

##### Laser Beam Alignment

Within the time gap between the tracking and positioning of the profiler, the base station needs to establish the communication link by aligning the laser beam with the photosensitive receiver on the profiler. Beam alignment is performed via interpolation. In terms of the laser-tracking angles corresponding to the three CCRs, $\Theta_{\mathrm{inq},1}$,$\Theta_{\mathrm{inq},2}$, and $\Theta_{\mathrm{inq},3}$, the angle corresponding to the photosensitive receiver $\Theta_{\mathrm{inq},4,t}=(\psi_{\mathrm{inq},4,t},\theta_{\mathrm{inq},4,t})$ can be calculated as:

(39) $$\{\begin{array}{c} \psi_{\mathrm{inq},4,t}=\frac{\left(L_{r,4}-L_{r,2})(L_{r,4}-L_{r,3}\right)}{\left(L_{r,1}-L_{r,2})(L_{r,1}-L_{r,3}\right)}\psi_{\mathrm{inq},1,t}+\frac{\left(L_{r,4}-L_{r,1})(L_{r,4}-L_{r,3}\right)}{\left(L_{r,2}-L_{r,1})(L_{r,2}-L_{r,3}\right)}\psi_{\mathrm{inq},2,t}+\frac{\left(L_{r,4}-L_{r,1})(L_{r,4}-L_{r,2}\right)}{\left(L_{r,3}-L_{r,1})(L_{r,3}-L_{r,2}\right)}\psi_{\mathrm{inq},3,t}\\ \theta_{\mathrm{inq},4,t}=\frac{\left(L_{r,4}-L_{r,2})(L_{r,4}-L_{r,3}\right)}{\left(L_{r,1}-L_{r,2})(L_{r,1}-L_{r,3}\right)}\theta_{\mathrm{inq},1,t}+\frac{\left(L_{r,4}-L_{r,1})(L_{r,4}-L_{r,3}\right)}{\left(L_{r,2}-L_{r,1})(L_{r,2}-L_{r,3}\right)}\theta_{\mathrm{inq},2,t}+\frac{\left(L_{r,4}-L_{r,1})(L_{r,4}-L_{r,2}\right)}{\left(L_{r,3}-L_{r,1})(L_{r,3}-L_{r,2}\right)}\theta_{\mathrm{inq},3,t} \end{array}$$

where $\Theta_{\mathrm{inq},1}=(\psi_{\mathrm{inq},1},\theta_{\mathrm{inq},1})$,$\Theta_{\mathrm{inq},2}=(\psi_{\mathrm{inq},2},\theta_{\mathrm{inq},2})$, and $\Theta_{\mathrm{inq},3}=(\psi_{\mathrm{inq},3},\theta_{\mathrm{inq},3})$ are the tracking angles corresponding to CCRs 1#, 2#, and 3#, respectively.

##### Laser Encoding

When the laser beam is aligned with the photosensitive receiver on the profiler, the base-station laser needs to modulate its light intensity. We emphasize that the profiler and the base station must be synchronized by a high-precision clock chip before the laser coding.

Encoding is accomplished via carrierless amplitude modulation (CAM): when the bit is 1 or 0, the base station transmits or turns off the laser light, respectively. The bit is dictated by the intensity of the light reaching the photosensitive receiver at the receiving end (1 when the intensity exceeds the threshold, and 0 when it is below the threshold).

When a downlink communication is required during the tracking process, the inquiry laser of the base station first aligns the laser beam with the photosensitive receiver on the profiler using Equation (39) and then modulates the laser light via the CAM code at the agreed synchronization time-point. Finally, the profiler end demodulates the light intensity of the photosensitive receiver to achieve downlink communication.

#### 3.5.2. Uplink

As described in Section 2.2, an MRR is located at position $p_{r,5}=(0,0,L_{r,5})$ of the profiler in the body coordinate system. The MRR modulates the reflected laser for communicating with the base station. The uplink communication process is similar to downlink communication, involving laser beam alignment and encoding.

##### Laser Beam Alignment

The beam alignment in uplink communication is similar to that in downlink communication. The laser beam angle corresponding to the MMR on the profiler $\Theta_{\mathrm{inq},5,t}=(\psi_{\mathrm{inq},5,t},\theta_{\mathrm{inq},5,t})$ is obtained via interpolation as follows:

(40) $$\{\begin{array}{c} \psi_{\mathrm{inq},5,t}=\frac{\left(L_{r,5}-L_{r,2})(L_{r,5}-L_{r,3}\right)}{\left(L_{r,1}-L_{r,2})(L_{r,1}-L_{r,3}\right)}\psi_{\mathrm{inq},1,t}+\frac{\left(L_{r,5}-L_{r,1})(L_{r,5}-L_{r,3}\right)}{\left(L_{r,2}-L_{r,1})(L_{r,2}-L_{r,3}\right)}\psi_{\mathrm{inq},2,t}+\frac{\left(L_{r,5}-L_{r,1})(L_{r,5}-L_{r,2}\right)}{\left(L_{r,3}-L_{r,1})(L_{r,3}-L_{r,2}\right)}\psi_{\mathrm{inq},3,t}\\ \theta_{\mathrm{inq},5,t}=\frac{\left(L_{r,5}-L_{r,2})(L_{r,5}-L_{r,3}\right)}{\left(L_{r,1}-L_{r,2})(L_{r,1}-L_{r,3}\right)}\theta_{\mathrm{inq},1,t}+\frac{\left(L_{r,5}-L_{r,1})(L_{r,5}-L_{r,3}\right)}{\left(L_{r,2}-L_{r,1})(L_{r,2}-L_{r,3}\right)}\theta_{\mathrm{inq},2,t}+\frac{\left(L_{r,5}-L_{r,1})(L_{r,5}-L_{r,2}\right)}{\left(L_{r,3}-L_{r,1})(L_{r,3}-L_{r,2}\right)}\theta_{\mathrm{inq},3,t} \end{array}$$

##### Reflected Light Encoding

During uplink communication, the base station laser first aligns the MRR using Equation (40). The profiler encodes the reflected light intensity by voltage-controlling the PDLC transmittance by the CAM method. When the bit is 1, the PDLC is powered on and non-opaque, so the light can be retro-reflected to the CCD camera on the base station. When the bit is 0, the PDLC is powered down and opaque, and the CCD camera cannot receive the reflected laser. The bit value (1 or 0) is decided by the CCD camera based on the received light intensity.

## 4. Experimental Results

To conveniently verify the feasibility of the proposed tracking, localization, and communication approach in the laboratory, we constructed a simplified version of the profiler module. The module was evaluated via two tracking and localization experiments and one preliminary communication experiment.

### 4.1. Tracking and Localization Experiments

**Figure 16:**
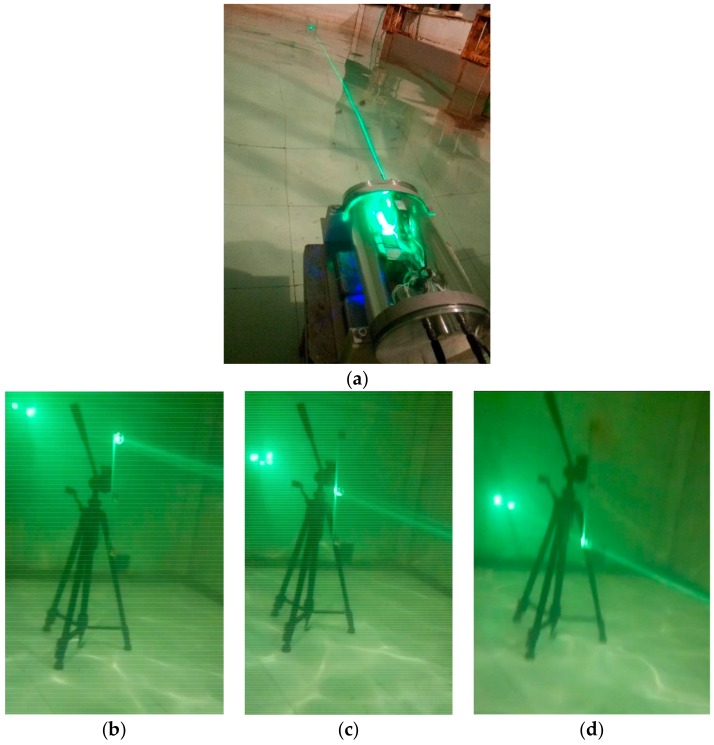
Tracking and localization experiment in the pool. (**a**) The base station emits laser beams and tracks the CRR; Bottom panels show the laser-tracking links of (**b**) 1# CCR (top CCR); (**c**) 2# CCR (middle CCR); and (**d**) 3# CCR (bottom CRR).

**Figure 17:**
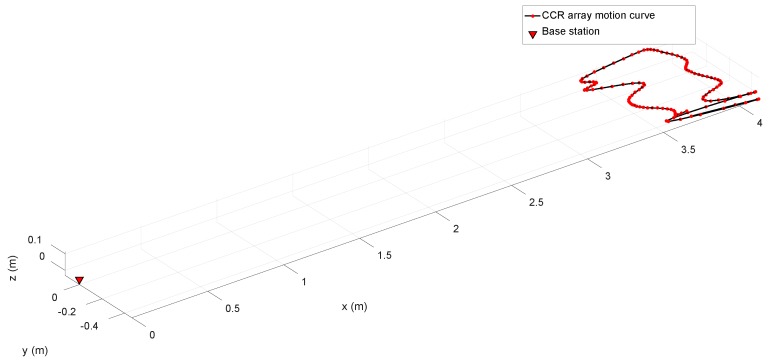
Experimental tracking and localization results.

The performance of the tracking and localization method was first determined in a basic tracking test performed in a 4-m-long pool. The base station and a CCR array were placed at opposite ends of the pool (see Figure 16), and the CCR array was moved at approximately 0.2 m/s (the approximate speed of a real HUP). Using the proposed method, the base station was required to track and locate the CCR array. The tracking and localization results are shown in Figure 17. The results confirmed that the base station can effectively track and locate the CCR array.

**Figure 18:**
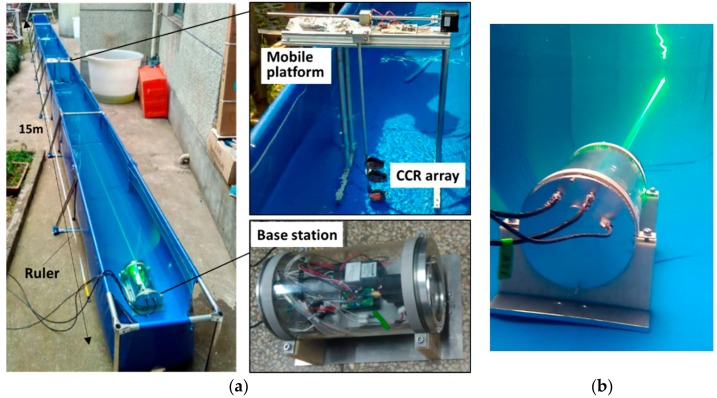
Tracking and ranging experiment in the long pool. (**a**) Photograph of the long-pool experiment; (**b**) base station tracking the mobile platform in the experiment.

To verify long-distance tracking and localization by the proposed method, the distance was measured in a 15-m-long pool as shown in Figure 18. The profiler end was replaced by a CCR array installed on a mobile platform.

**Figure 19:**
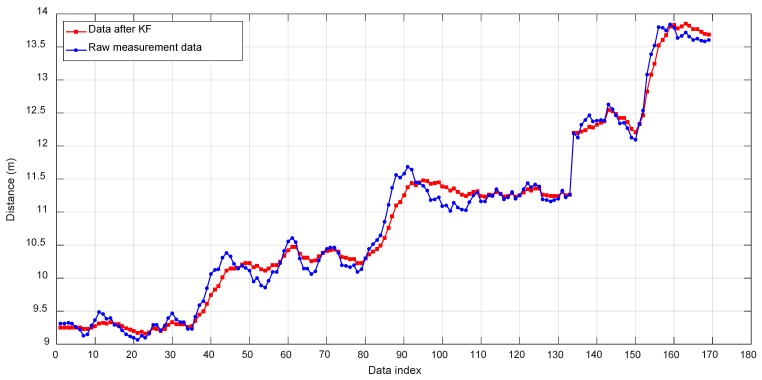
Tracking and ranging results of the long-pool experiment.

The performance of the localization approach was tested at five points located at 9.25, 10.25, 11.25, 12.25, and 13.6 m from the base station. After the base station completed initialization, the mobile platform was sequentially moved to each of the five points, and it stayed there for a period of time. Figure 19 shows the raw measurement data and the data after Kalman filtering collected during the experiment. The mean values of the KF-filtered data at the five points are listed in Table 2. The localization error is 0.165 m at 13.6 m and the error rate is 1.21%. The localization error decreases with the decrease of the distance. The error of the nearest localization distance at 9.25 m is 0.062 m, and the error rate is 0.67%. It can be seen from the test results that the tracking algorithm is effective and achieves a high localization accuracy.

### 4.2. Preliminary Communication Experiment

**Figure 20:**
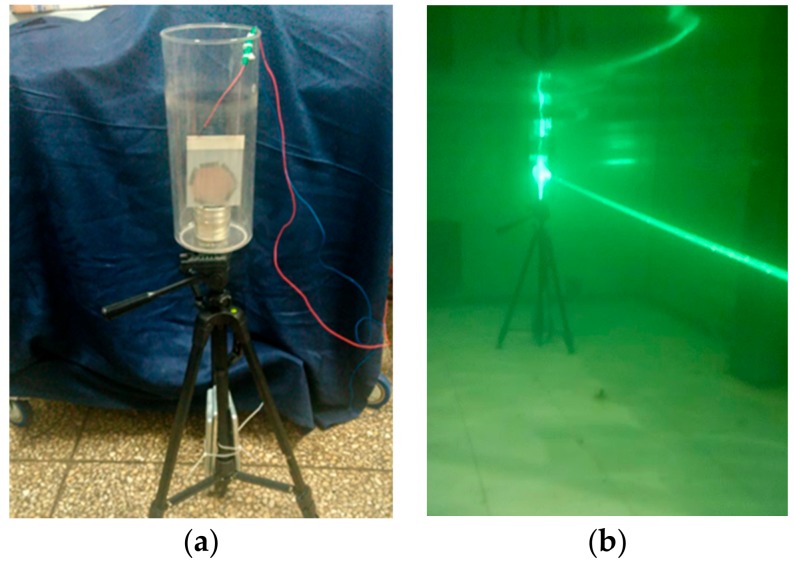
Preliminary communication experiment. (**a**) The MRR used in the experiment, which contains a CCR behind a PDLC film; (**b**). MRR operating during the experiment.

To preliminarily verify the feasibility of reflective (uplink) communication by the proposed method, an initial test was conducted in the 4-m-long pool. As shown in Figure 20, an MRR comprising a CCR and a PDLC film was placed at one end of the pool.

**Figure 21:**
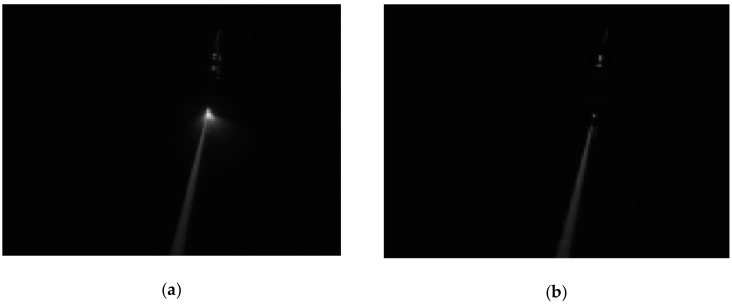
Images received by the CCD camera during the underwater reflective communication test: (**a**) high-level modulation image and (**b**) low-level modulation image.

**Figure 22:**
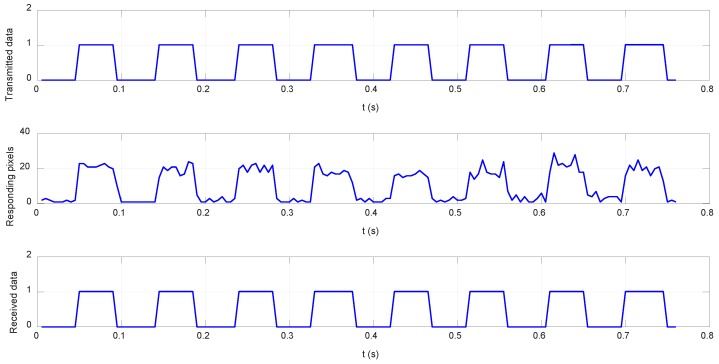
Results of the reflective communication experiment.

During the experiments, the MRR was switched at 20 Hz. The light-intensity sampling frequency and exposure time of the CCD camera at the base station were 200 Hz and 500 μs, respectively. Sampled raw pictures at high-level and low-level modulation are shown in Figure 21. Here, the light-intensity threshold of the CCD camera was set to 150. Figure 22 shows the output of the MRR and the data received by the camera of the base station. The base station well received the information modulated by the MRR.

## 5. Conclusions and Future Work

Conventional acoustic methods are not straightforwardly applicable to localization and communication of underwater vehicles in close-range and near-seabed situations. Therefore, we proposed a laser reflection- based localization and communication method with low power consumption, low cost, and high resolution. In this paper, an underwater base station is connected to an observation network on the seabed for locating and communicating with a HUP, and a matching reflection module is mounted on the HUP. The base station tracks the HUP by laser light emission. The reflector reflects and modulates the incident laser with very low power consumption.

A particle filter-based approach was proposed for tracking the HUPs. This process includes a priori prediction and sampling based on the previous cycle, and importance resampling based on the sampled response. The HUP’s location is coarsely determined via a geometric calculation with refractive compensation, and finely determined via a nonlinear optimization method based on the objective function obtained by the least-squares method. The localization accuracy is further improved by Kalman filtering. The effectiveness of the filtering method was first verified in simulation results. To recover any link failure during tracking, we developed a tracking state discrimination method based on K-means clustering, which restores the link via linear interpolation. The interpolation method also aligns the corresponding module when the base station communicates with the HUP. After alignment, the communication is encoded using the CAM method.

The feasibility and performance of the proposed tracking and localization methods were evaluated in two experiments. The base station effectively and stably tracked the moving reflector and performed accurate localization. The positioning error at the farthest localization distance of 13.6 m in the experiment was 0.165 m, and the localization accuracy increased as the distance decreased. The communication methods of the proposed system were then verified in a small pool. The CAM encoding method was used to convey complete information to the reflective end, enabling the base station to receive all information and implement the uplink communication.

In future work, we will first conduct a field trial over a longer distance to verify the effective range of the system. After that, we will integrate the retroreflector module into the existing HUP, and deploy the base station in the South China Sea, where it will be connected to the existing seabed-observation network. This deployment will fully verify the proposed tracking, localization, and communication methods.

## Figures and Tables

**Table 1:** Comparison of the fine localization errors with and without Kalman filtering.

Method	RMSE in $x_{\mathrm{base}}$ (m)	RMSE in $y_{\mathrm{base}}$ (m)	RMSE in $z_{\mathrm{base}}$ (m)
Fine localization	0.287	0.014	0.014
Fine localization + Kalman filter	0.087	0.006	0.006

**Table 2:** Comparison between the actual distance and the measurement data.

Actual Distance (mm)	Measurement Data Mean (mm)	RSME (mm)	Error Ratio
9250	9271	62	0.67%
10,250	10,293	81	0.79%
11,250	11,322	102	0.90%
12,250	12,352	139	1.13%
13,600	13,740	165	1.21%
